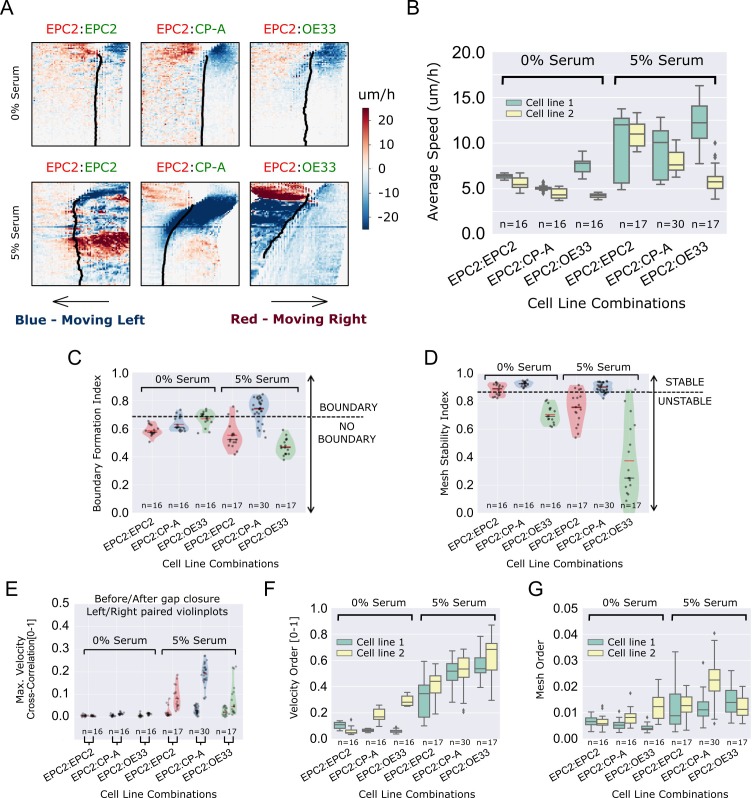# Correction: Motion sensing superpixels (MOSES) is a systematic computational framework to quantify and discover cellular motion phenotypes

**DOI:** 10.7554/eLife.49823

**Published:** 2019-07-02

**Authors:** Felix Yuran Zhou, Carlos Ruiz-Puig, Richard P Owen, Michael J White, Jens Rittscher, Xin Lu

Matson JP, Dumitru R, Zhou FY, Ruiz-Puig C, Owen RP, White MJ, Rittscher J, Lu X. 2019. Motion sensing superpixels (MOSES) is a systematic computational framework to quantify and discover cellular motion phenotypes. *eLife* **8**:e40162. doi: 10.7554/eLife.40162.

Published 26, February 2019

During final figure preparation the similar velocity kymographs for 0% serum EPC2:CP-A and EPC2:OE33 in Figure 3A were switched. This mistake was noticed by the authors when rereading the published version. Also in Figure 3A, the red and green font colours used for the cell line names were accidentally switched; these colours should match those in Figure 1 as the same example videos were used for both figures. Figure 3A has been corrected accordingly. These corrections do not affect any of the scientific findings or conclusions of the original paper.

The corrected Figure 3 is shown here:

**Figure 1:**
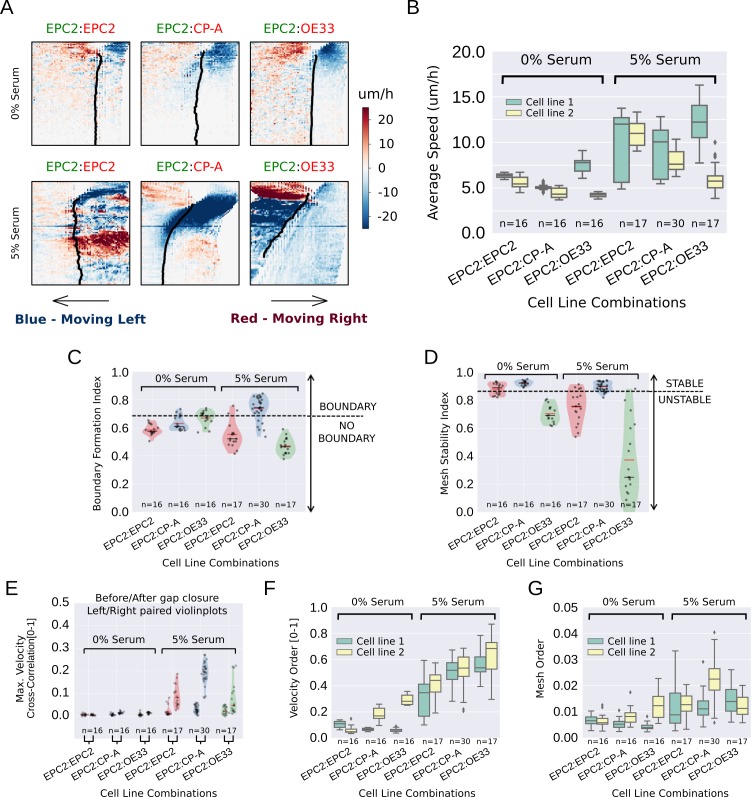


The original Figure 3 is shown here for reference:

**Figure 2:**