# Supplementary material for: Motion sensing superpixels (MOSES) is a systematic computational framework to quantify and discover cellular motion phenotypes
Source: eLife. 2019 Feb 26;8:e40162. doi: 10.7554/eLife.40162 (PMC6391079; doi:10.7554/eLife.40162)
Supplement: Supplementary file 1. [file elife-40162-supp1.docx]

|  |  | **Exp 1 (n=37)** | **Exp2**  **(n=50)** | **Exp3**  **(n=21)** | **Exp4**  **(n=17)** |
| --- | --- | --- | --- | --- | --- |
| **Media**  **Condition** | **Cell Combinations** | **4x, 10 µM dye, asymmetric divider** | **4x, 10 µM dye, symmetric divider** | **4x, 10 µM dye, symmetric divider** | **2x, 2.5 µM dye, symmetric divider** |
| **5% serum** | **EPC2:EPC2** | **3** | **5** | **7** | **2** |
|  | **EPC2:CP-A** | **5** | **8** | **13** | **4** |
|  | **EPC2:OE33** | **5** | **7** | **1** | **4** |
|  | **EPC2:AGS** | **-** | **-** | **-** | **2** |
|  | **CP-A:CP-A** | **-** | **-** | **-** | **2** |
|  | **CP-A:OE33** | **6** | **-** | **-** | **-** |
|  | **OE33:OE33** | **-** | **-** | **-** | **3** |
| **0% serum** | **EPC2:EPC2** | **6** | **10** | **-** | **-** |
|  | **EPC2:CP-A** | **6** | **10** | **-** | **-** |
|  | **EPC2:OE33** | **6** | **10** | **-** | **-** |
