# Supplementary material for: Motion sensing superpixels (MOSES) is a systematic computational framework to quantify and discover cellular motion phenotypes
Source: eLife. 2019 Feb 26;8:e40162. doi: 10.7554/eLife.40162 (PMC6391079; doi:10.7554/eLife.40162)
Supplement: Supplementary file 2. [file elife-40162-supp2.docx]

| **All were imaged 2x, 2.5 µM dye, symmetric divider, total = 65 videos** | | | | | | |
| --- | --- | --- | --- | --- | --- | --- |
| **Experiment No.** | **Media conditions** | **EGF Concentration** | | | | |
|  |  | **0 ng/ml** | **2.5 ng/ml** | **5 ng/ml** | **10 ng/ml** | **20 ng/ml** |
| **1** | **5% serum** | **-** | **-** | **2** | **2** | **1** |
|  | **0% serum** | **2** | **-** | **2** | **1** | **-** |
| **2** | **5% serum** | **4** | **4** | **4** | **4** | **4** |
| **3** | **5% serum** | **4** | **-** | **4** | **3** | **4** |
| **4** | **0% serum** | **4** | **4** | **4** | **4** | **4** |
